# Mountains of Gold: The Alpine Vaults of the Swiss National Bank, 1939–46

**DOI:** 10.1177/00220094241306985

**Published:** 2025-02-08

**Authors:** Ludo Groen

**Affiliations:** Institute for the History and Theory of Architecture (gta), ETH Zurich, Zurich, Switzerland

**Keywords:** Alps, gold, infrastructure, Second World War, Swiss banking, Switzerland

## Abstract

It is widely believed that banks in Switzerland keep their hoards of gold safely in vaults in the city, but during the Second World War, the banks discovered a more efficient, secure, and spacious place for their gold: the Alps. Using built objects as evidence, this article describes how, in 1939, a military ammunition depot in the Bernese Alps was converted into a mountain vault, for the Swiss National Bank to store its domestic gold reserves. What started as an evacuation site, along the way changed purpose from protection against the enemy to catering to them. As the gold reserves of the National Bank grew, its vaults in Bern and Zurich were no longer large enough to store all the incoming precious metals. Only by its decentralization to the Alps could Switzerland keep up the gold trade with, amongst others, Nazi Germany. Based on extensive research in the Swiss National Bank's archives, the article discloses, for the first time, evidence of the wide-ranging infrastructures and bureaucracies that facilitated the storage of gold in the Alps – a history that until today has been dominated by popular myths rather than critical historical inquiry.

Behind painted surfaces of concrete and steel, resembling the mottling of adjacent rocks, six ammunition depots were recessed into the mountain. The autumn wind had blown the cover off the site's natural camouflage that morning in 1939, when Swiss National Bank Director Max Schwab, its Treasurer Erich Blumer, and an engineer by the name of Friedrich Lienard arrived in Interlaken, a tourist town at the foot of Switzerland's Bernese Alps. After what must have been an amicable hour's drive, the suited men clambered out of the bank's black Buick and were saluted by a uniformed colonel, major, captain, and lieutenant of the Swiss Army. In front of the depots, conspicuously stretching over 100 m along the shores of Lake Thun, was a railway track recessed into the asphalt, parallel to an elevated loading platform. From there, a blast door swung open into a narrow corridor, at the other end of which was a room that had been cleared for the National Bank. Treasurer Blumer scribbled the room's dimensions of 8.5 m by 7 m in his squared notebook, which, on ordinary office days, he used to draw spreadsheets and graphs. Back at the bank, he must have ripped off the paper, handed it to his secretary, and started dictating captions like ‘the floor plan of the room available to us is roughly as follows’, and ‘here, a 50 cm thick reinforced separation wall, connected to the rock’ (see Figure 1). Like an architect signing his drawing, Blumer signed the note with ‘Bl’ in blue pencil at the bottom right, the same signature that authorized the Swiss banknotes. The resulting bedraggled piece of paper is the first, and about the only, floor plan of a mountain vault in the National Bank's archives. The absence of such paper records can be related to the fact that, until today, the writing of the histories of Swiss banks is still dominated by popular myths rather than critical historical inquiry.^1^

**Figure 1.**
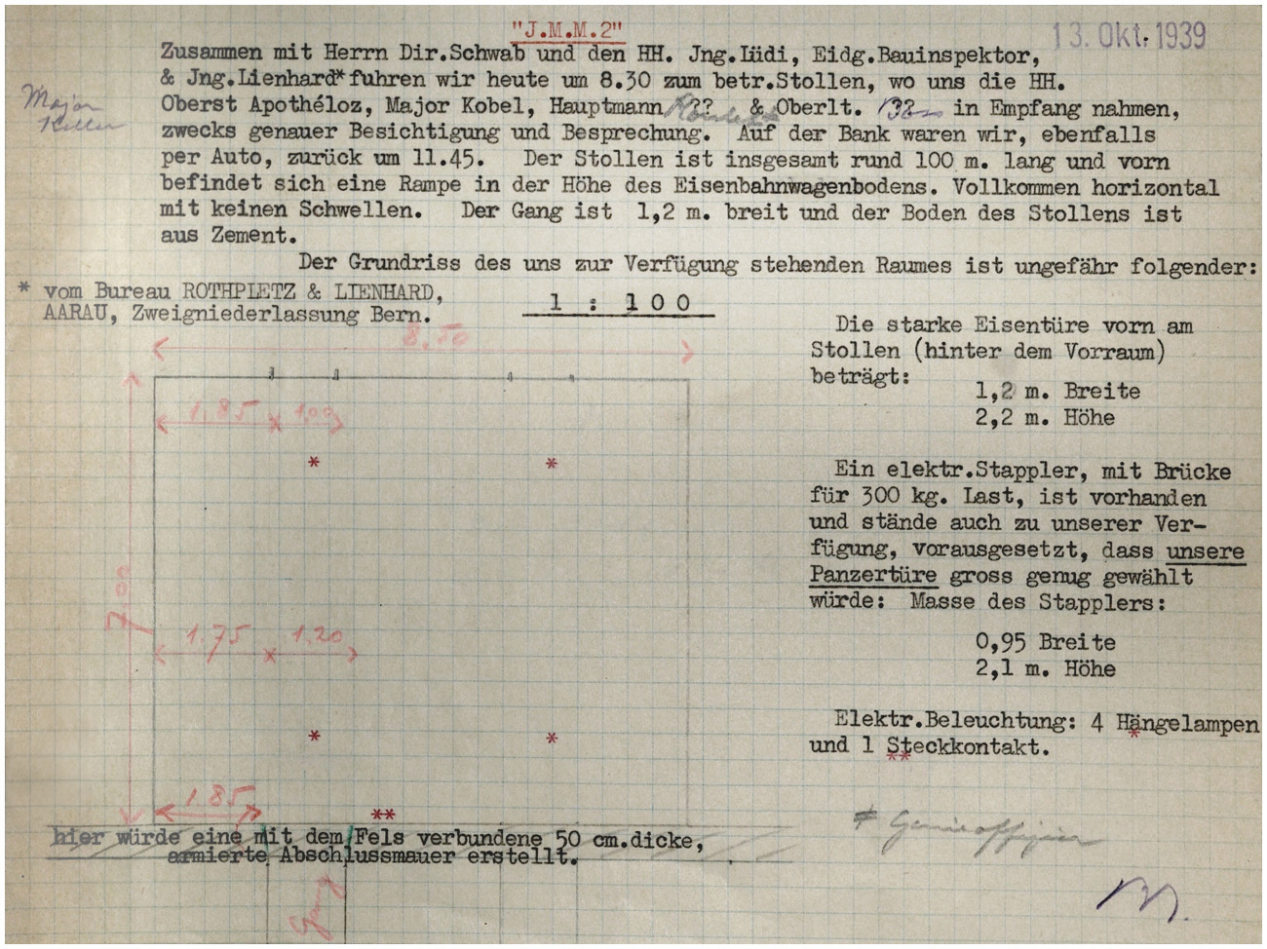
Drawing of underground cavern as sketched by National Bank Treasurer Blumer on 13 October 1939. Source: SNB Archive, signature 1,000,971.

The day was 13 October 1939, six weeks after Nazi Germany's invasion of Poland that marked the beginning of the Second World War. In anticipation of dark times, the Swiss Army's commander for the city of Bern had expressed his concerns about the safety of the nation's gold reserves vaulted below the bank on the Bundesplatz, believing that the building was well protected against burglary and fire, but that if the federal city were shelled, ‘these great reserves of our country would be destroyed by one blow’.^2^ To this end, in 1938 the National Bank built its first mountain depot in Andermatt, on top of the Gotthard railway tunnel, where it moved two-thirds of the bank's gold.^3^ By the fall of 1939, the newly appointed General Henri Guisan demanded a geographical redistribution of the gold across the country's security zone in the Alps, on suitable locations from both military and logistical point of view. Considering the military sites that could be vacated at short notice, the General Staff proposed that one of its newest ammunition depots outside Interlaken be made available. That fall also Credit Suisse, Switzerland's largest commercial bank at the time, had converted a grand hotel in Interlaken, to set up office and store its gold and other valuables.^4^ Following their example, the National Bank agreed to the terms of tenancy for the ammunition depot (there was no rent, only a reimbursement of energy costs), signed the lease, and codenamed the cavern as ‘JMM2’, an abbreviation for ‘Interlaken Munition Magazine 2’. Remarkably, in its classified correspondence, the bank did not refer to the depot as a *tresor*, as they called the urban vaults in Bern and Zurich, but continued to call it an ammunition magazine, contributing to the room's perpetual clandestine character, not without reason.

Engineer Lienhard knew the cavern like no other. He and his colleague Ferdinand Rothpletz had designed it a few years earlier to store 450 railway wagons of heavy infantry weapons. At the time, it was not possible to store these explosives, unprecedented in their powerfulness, in regular air raid shelters, so the country's most renowned tunnel builders were commissioned to design a depot that could safely store this load.^5^ The engineers found a rugged edge of a nearby forest, half a kilometre outside Interlaken, in the hamlet of Lütscheren.^6^ The place still retained its quiddity those days, a stony terrain that even the fir trees found arduous to inhabit. A passing railway, canal, and road coming from Interlaken made the site convenient for cargo, but also meant that the project did not go unnoticed by others. Once the excavation began, idle chatter spread through the town, eventually reaching the national quality newspaper *Der Bund*. Carefully archived in the depot's construction dossier, a short article entitled ‘Benevolence, Carelessness, or What?’ wondered aloud:

In the middle of the touristic area of the Bernese Oberland, directly on a busy road, not far from a major tourist resort, distinct constructions are currently being built to safeguard our national defence. Thousands of foreign cars pass this place during the season. Photographs can be taken from cars, trains or by walkers without any obstruction, because there is no barrier, the construction site is entirely visible. The site can be marked with precision on any map which, as far as I know, is available in any bookshop, even to strangers. The only thing missing is the affiche that invites free viewing. But we are a neutral country to which no one is doing any harm! One really has to ask oneself whether we Swiss are so carefree, so benevolent or so simple-minded (just shepherd boys) that such a thing is possible.^7^

The outcry was sparked by an unannounced site visit from two Englishmen. It was on a spring Sunday when the duo, allegedly dressed as officers, arrived. A civilian guard in front of the construction site initially forbade them entry, but after generously tipping the guard he turned a blind eye so that the two men could have a look inside. In shock that the military had not cordoned off the place, the local population must have leaked the incident to the press. The office of the attorney general investigated the case, and in their matter-of-factness, established that the single guardian was only there because on Sundays the accident insurance did not cover liability claims resulting from falling stones (natural disasters were seen as liability, not intruders), and that during working days the site was in fact freely accessible. The contractor had some serious explaining to do and denied that his guard had allowed anyone to access, or accepted a bribe of any kind. The responsible engineer added that during an unannounced Ascension Day visit, he indeed saw two elderly English gentlemen climb the ramp, and ordered them to leave before they could enter the first ammunition chamber. The two men, who according to him did not look like officers at all, were not noticed by the guard, who happened to be in the site hut, enjoying the break he was entitled to.^8^ The absurdity of the story helped to pass it off as a fable, and the two Englishmen were dismissed as lost hikers in red-laced mountaineering boots. The only action taken was to order the renegade railway conductors, who were drawing the attention of passengers to the construction site when rolling past, to put a stop to this nonsense.

Half a year after the celebratory vernissage of the ammunition depots, engineer Lienhard was invited back to the site for the morning visit described above, to investigate the repurposing of one of its six rooms. Two extra pendant lights with separate switches had to be added so that the auditors had sufficient light to regularly check-in on the gold and a half-metre thick concrete wall, reinforced with 2.5 tons of steel, had to separate the vault from an anteroom and provide a structure for an armoured vault door.^9^ The latter was ordered by the treasurer himself, because only a handful of Swiss suppliers met the bank's highest standards. His vigilance to take care of the door himself proved wise, as three years later, the military by chance discovered that all the locks on the outer doors of the other ammunition depots had a mechanical defect, so that they could be opened without much effort with an ordinary square key.^10^ The ventilation system also had to be upgraded for its new occupant, to avoid condensation. The precious metals required an even greater supply of fresh air than ammunition or humans. Even though pure gold does not rust, the 99.9 per cent purity of the gold bars was no guarantee to prevent rust and tarnish over time. An axial ventilator was installed with the capacity equal to the contemporary ventilation standards of a 30-person office and with an armoured valve that could be closed in the event of a gas attack (Figure 2).^11^

**Figure 2.**
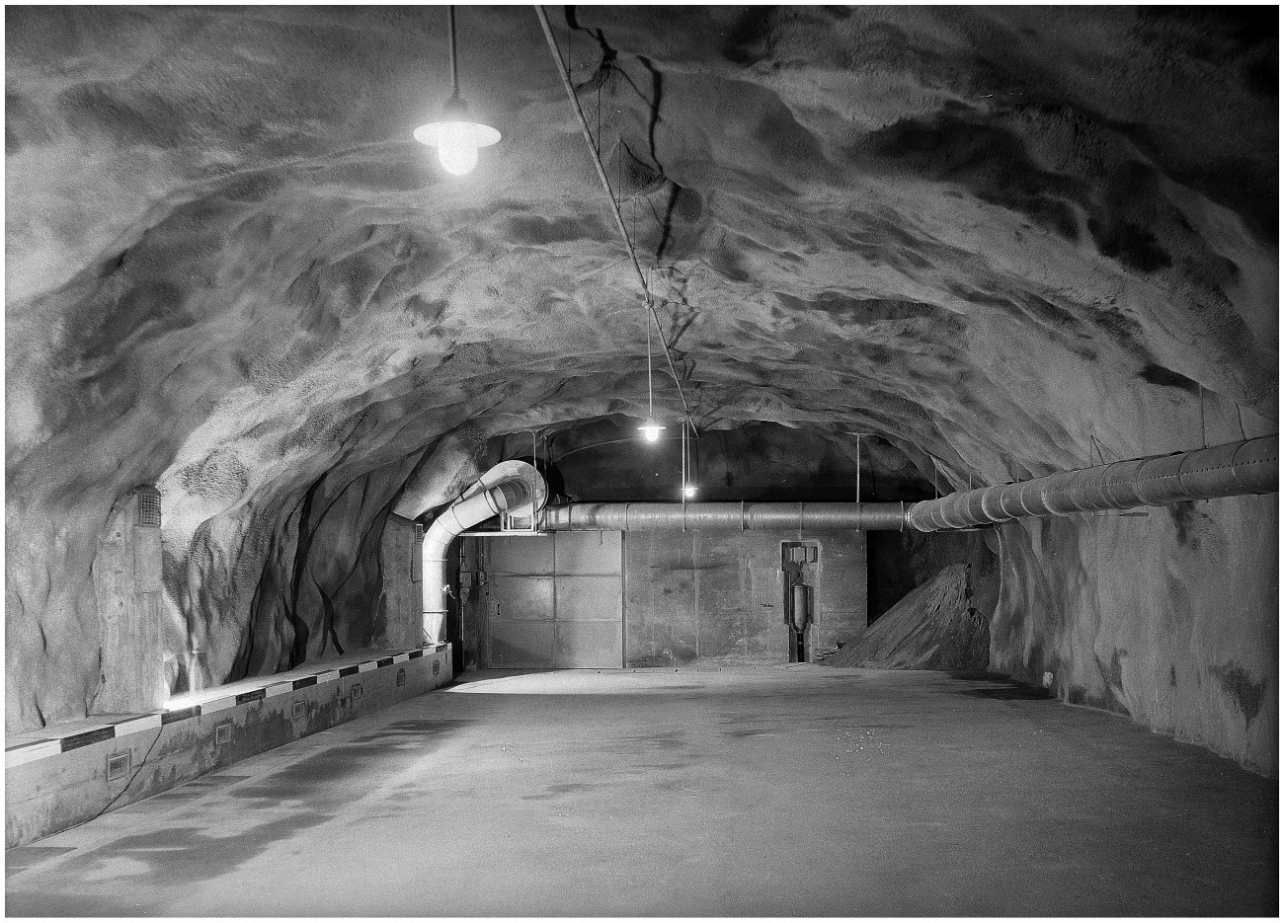
Photograph of an underground cavern in Amsteg, including ventilation system, comparable to the depot in Interlaken. . The minutes of a meeting of the bank's directors of the Swiss National Bank from 25 August 1960 report that between 1941 and 1945 also this site in Amsteg was used for the storage of gold bars, coins, and banknotes. Swiss National Bank Archive, signature 1,000,970. Source: Swiss Federal Archives, signature E5792#1988/204.

While the finishing touches were being put on the conversion of the Interlaken depot, General Guisan ordered in the autumn of 1939 that a third evacuation site be found in the west of the country, in the upper Simmen valley or in the neigbouring Gruyere area of the canton Fribourg (see Figure 3). This would make occasional relocations of the gold possible depending on the changing circumstances. Guisan and his staff even advised the bank on numbers: around CHF200  m could remain in the Gotthard, up to 500 m had to move to Interlaken, and another 300 m or more to third, to be found site. And so it happened that another of the bank's directors, Paul Rossy, who grew up close by, was sent on a reconnaissance trip, joined again by engineer Lienhard, to picture any necessary constructional precautions on the spot. An acquaintance of the bank had pointed out that the Carthusian monastery of La Valsainte would be excellently suited as an evacuation site, and that provisions had already been made there by the Cantonal Bank of Fribourg.^12^ On a drizzly November morning, the men left Bern with military transport. Driving down to Bulle, south of Fribourg, a district officer awaited them to guide the way to the outlying monastery, climbing to an altitude of 1015 m. Their contact at Fribourg's cantonal bank had kindly telephoned La Valsainte to announce their visit and provided an introduction letter to the monastery's prior. Branching off the main road to Jaun into a secluded side valley of the Lake Montsalvens, numerous hairpin turns and bridge crossings later the monastery finally appeared between the autumn-gradiented hills. The settlement resembled a carefully planned small village, cloistered by a heavy stone wall, and centered around a church. The banker and engineer were received by the Carthusian monks in the most courteous manner before they were directed to the convent's large cellars. From the lower side of the garden, two underground corridors extended over 190 m, one of them even equipped with a roller conveyor belt, from where side corridors led to the cellars. Unfortunately for the National Bank, all these rooms were already taken by the cantonal bank, who transformed them with armoured doors and shelves into small tresors.^13^ The prior could only offer an 80-m long side passage that was rather narrow, not easily accessible, and most of all very damp. Another problem was the unpaved road to the monastery, which was fairly accessible in summer and autumn, but in winter the goat path disappeared in the snow so that a gold transport would have to take place with horse-drawn sleighs.^14^

**Figure 3.**
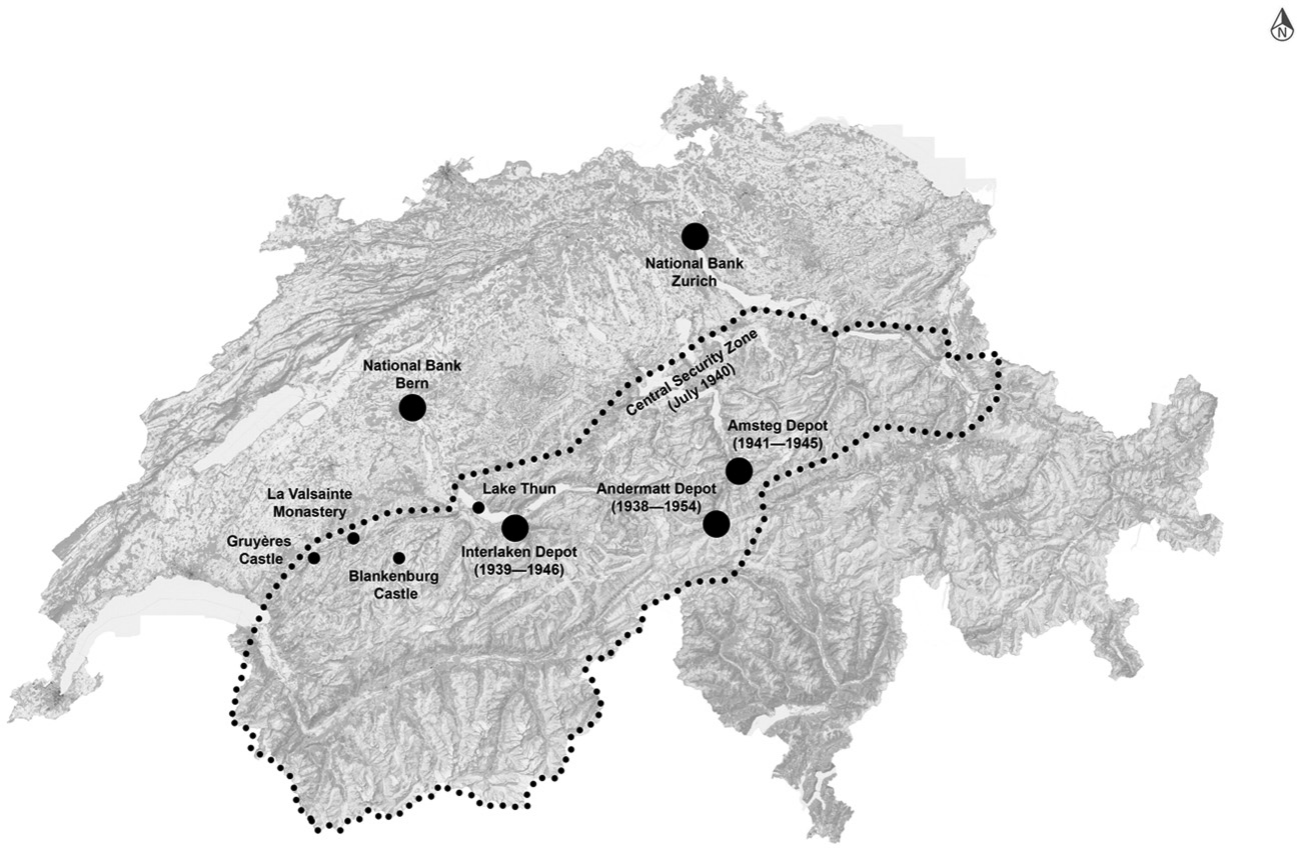
Map of the geographical distribution of gold depots of the Swiss National Bank during the Second World War. In large dots, the depots that were in use, in small dots the considered but not used sites. The dotted line demarcates the central security zone, as stipulated in the Swiss Army's Operation Order 11 of 12 July 1940. Map by author. Source (underlayer): Swisstopo, Topographic Landscape Model (Bern 2024).

Daunted by the wintry prospect, Director Rossy and engineer Lienhard continued their journey to the Gruyères Castle, a square-planned fortification with remains from the thirteenth century, retreated to the top of a grassy hill.^15^ The scaffolded castle was recently sold to the canton and local craftsmen were busy carrying out the necessary conservation work on its foundations and walls. Once completed, the National Bank could potentially give it a new public duty. Gold-loaded trucks entering through the castles’ entrance porch would be able to drive straight into the courtyard from where, some steps down on the right side, there was a storage cellar of 10 by 7 m. Masonry walls of up to 2 m thick and a vaulted ceiling guaranteed a safe perimeter. A narrow embrasure window would have to be obstructed with two heavy lattices and the entrance secured with an armoured door. Afterwards, its 70 square metres could safeguard around CHF500 m in gold bars and coins. What concerned the visitors though, was the castle's guarding, as its mere resident was a lonely gatekeeper lodging in one of its outbuildings. And only a single policeman was stationed in the nearby village of Gruyères, meaning that a permanent and costly military guard would have to be installed, housed, and fed in the castle.

The itinerary's final stop was Blankenburg Castle, another mediaeval settlement of national significance confined to a gentle hill. In the three former prisoner cells of 3 by 3 m, the bank could store gold up to CHF300 m. Once again, the small embrasure window opening had to be secured, an armoured door with a lock mounted, and electric lighting extended. More challenging was the room's supply route, from the forecourt via an open staircase to the inner upper courtyard where another stone staircase led to the basements. A conveyor belt or elevator could overcome this hurdle but would be prohibitively expensive. Next to the rooms that were on offer, the insurance company La Genevoise already set up an evacuation room here for its security papers, and as the castle also served as a seat of the Bernese court, the district police was based in an adjacent house, making the castle's guarding easier to solve.^16^

That not only conventional options were considered to hide 250 tons of gold from an invading enemy becomes evident from the very last option contemplated by Director Rossy: the sinking of gold batches to the beds of one of Switzerland's lakes.^17^ A year earlier, the contentious founder of Migros, Gottlieb Duttweiler, had presented a plan to the federal government to guarantee food security in the dark times ahead. While warehouses were at risk of being bombed and expensive to build, Duttweiler proposed to sink barrelled grain, oil, and fuel in centrally located lakes. In the summer of 1939, his first experiments took place in Lake Thun, as it happened right in front of the National Bank's gold depot in Interlaken.^18^ . The conservative bankers realized that such a solution would indeed make the question of security much easier, but that the gold's weight required much more advanced technical equipment for its dumping and lifting than the barrels of grain, hindering a rapid removal in the case of an emergency. During the car ride back home, not entirely convinced by what they had seen that day, the monasteries, castles, and lakes were all discarded by Director Rossy and his engineer Lienhard because guaranteeing their safety required much more costly interventions and policing than the repurposing a room in pre-existing military sites. For the time being, the National Bank stuck with their two gold depots in the Gotthard and Interlaken.

The winter's first snow had already whitened the Swiss capital's red roofs, when General Guisan in November 1939 ordered the removal of CHF700 m in gold bars and coins, of the total reserve of CHF1 billion, from the Gotthard gold depot to Interlaken as soon as the reconversion project was completed.^19^ That day in Bern, Treasurer Blumer's thoughts must have wandered back to the freezing nocturnal adventures he and his colleagues had endured just a year and a half earlier in order to bring those same bars and coins up to the vault in Andermatt's Fort Bühl, halfway the Gotthard Pass. There, it was thought, the gold would be evermore safe. But now that the ultimate responsibility for the matter rested no longer with the National Bank but with the newly appointed general, the bank had to plan its partial removal, even in the worst possible moment of the year. Anticipating January's inevitable heavy snowfall, they would have to cross the Schöllengorge leading to the vault with an excruciatingly slow cog railway. This meant that no less than three different railway companies had to be involved in the journey: first the Schöllenenbahn from Andermatt to Göschenen, from there the Federal Railways via Lucerne to Bern, and eventually the Lötschbergbahn down to Interlaken.^20^ Another complication was that by the fall of 1939, around 850,000 Swiss men were mobilized out of a population of 4 million, among them the majority of the National Bank staff. In view of the great risks involved in the transport and the utmost importance to guarantee its secrecy, Treasurer Blumer did not entrust the precious transport to the military. Only his own staff, who already carried out the Gotthard transports the year before, were aware of the perilous nature of the task and enjoyed his full confidence. Switzerland's militia system of an armed population, allowed the bank's chief treasurer, the chief auditor, and the chief clerk, and eight of their colleagues to change into field-grey uniforms, step into tall leather boots, put on a beret or cap (depending on their rank), take up arms, and serve the country simultaneously as bankers and soldiers. At the transhipment places, local garrisons would guard the loading and unloading, but only the six staff members (administered by five auditors) were supposed to touch the precious cargo and perform the arduous labour.^21^

With clockwork precision, Treasurer Blumer, who was in charge of the entire operation, laid out a seven-day program and reported on the efforts afterwards to the bank's directors. With a cold start, the treasury department arrived on Sunday 28 January 1940, delayed by 43 min as the train had to stop twice in the gorge to shovel away the fresh, impenetrable masses of snow. The snow-blanketed Fort Bühl laid peaceful in the gorge, cloaking in its depths the gold depot. After dining with the responsible officers, they bunked that night with the soldiers in the cold barracks. The next morning, rising early for their registration with the chief fortress officer, it was time to start the preparations. To ease the manual labour compared to last year, 218 platforms and 4 lift trucks were delivered.^22^ These tools, which only later became known as standardized pallets, were entirely new to Switzerland. Their designs crossed the Atlantic from the United States where Henry Ford had introduced them in his magazines to substitute the heavy lifting.^23^ For that same reason, Blumer had arranged for them to be delivered to Fort Bühl. The next day, another seven vigorous bankers arrived from Bern and Zurich to pack the 2402 boxes (each comprising four gold bars) and stack them onto the pallets. These were then driven by a pallet lift to a conveyor belt that crossed a 220-m long tunnel to the fortress’ exit in the gorge. Once leaving the mountain, the pallets were loaded onto small cog railway wagons. Even dinner was served that evening in the vault's anteroom, so that a little before midnight, the first 3 chock-full wagons crept in a snow-muffled ride down to Göschenen. On the slippery wooden railroad ties that formed the platform, the cargo had to be transhipped into the wagons of the Federal Railways, a laborious operation that was repeated that same night with another seven wagons under the watchful eye of the 9th division garrison.^24^

From 7:15 the next day, all activities were suspended as the railway resumed its regular timetable, giving the bankers the chance to rest all day in the barracks. After the dark set in, the packing of the wagons in the gorge continued. Pallets rolled loudly in and out of the tunnel, faintly lit by the stars against the night sky. At around 4:45 the next morning, the nocturnal silence returned as the last two wagons, including all staff, rattled down to Göschenen. After a well-earned breakfast at the station buffet, where by now they were frequent guests, 12 wagons (and a single second-class compartment) set off with 88 tons of gold, worth almost 1 per cent of Switzerland's GDP that year. To be as discreet as possible, the trip was listed as a special ammunition transport, without disclosing its point of departure and arrival. But during the lingering 6-h drive, there was no time to recover from the night shift. The carriers changed roles into guards and had to take notice of any extraordinary detail occurring around the wagons or the tracks. In Lucerne, they were startled for a moment when a spring bearing broke in the third-last wagon, but without much interruption, the journey could be continued.

Arriving in Interlaken after midday, only a couple of minutes behind schedule, they were awaited again at the station by the major, colonel, captain and their garrison. While the bankers enjoyed a hearty lunch, the soldiers drove the wagons in the direction of the cavern, so that when they finished their plates, unpacking could start right away. The sixteen local men in charge of its guarding sympathized with the carriers and helped with the final stretch, together emptying six wagons shortly after midnight. After spending the short night in the frosty second-class carriage, the next morning the last gold bars were moved inside the brand-new vault, the auditors completed the paperwork, tidied up the cavern and left, the heavy door clanging shut behind them.^25^ Field-grey-coated, Blumer rolled back into Bern's Hauptbahnhof that Saturday afternoon; black-coated he returned to the bank on Monday morning to dictate his adventures to his secretary. The dutiful man whose signature authorized the Swiss banknotes, who had spent a winter night in an unheated upholstered second-class carriage guarding the national treasures, was only modestly aware of seeing through another cold heroic act when he wrote: ‘this completes one more episode in our gold evacuation story, and I hope it was done to the satisfaction of our bank authorities’.^26^

**Figure 4.**
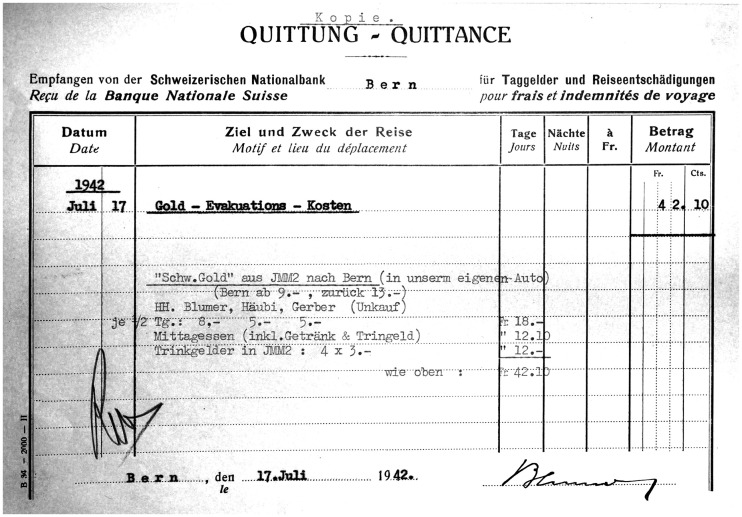
Travel declaration for a visit to the Interlaken depot JMM2, signed by Treasurer Blumer on 17 July 1942. Source: SNB Archive, signature 1,000,971.

Lunch at CHF12.10 including a drink and tip, CHF22 for the usage of the company car, plus a daily allowance per staff member – the declarations signed by Treasurer Blumer during the Second World War (Figure 4) scrupulously record the dates of his visits to the gold depot in Interlaken. Some light on the purpose of these visits was shed only half a century later by the Eizenstat report commissioned by the United States, disclosing that Swiss banks accepted looted gold throughout the war, and in this capacity bankrolled Nazi Germany.^27^ In even greater detail, the Swiss state-appointed Bergier Commission afterwards concluded that during this period, Switzerland purchased approximately CHF1.6 billion in gold from the German Reichsbank in exchange for Swiss francs. As the *Reichbank*'s gold coffers were empty at the dawn of the war, this included gold looted from Holocaust victims and expropriated from federal banks of occupied Austria, Czechoslovakia, Belgium, and the Netherlands. With the francs Nazi Germany received in return, they imported weapons, machinery, and chemicals, and other goods needed for the war economy.^28^

The frequency of Blumer's travel and lunch declarations corresponds to the five periods identified by the economic historians Vincent Crettol and Patrick Halbeisen in their analysis of the Swiss National Bank's incoming and outgoing gold transactions between 1939 and 1945.^29^ From the general mobilization in September 1939, the first period was characterized by a silence before the storm and left Switzerland wavering between states of fear and determination to face the enemy.^30^ Besides the earlier described redistribution of gold from the Gotthard to Interlaken, the wait-and-see stance triggered a capital flight to the United States with the National Bank shipping gold overseas from Bern via the ports of Genova, Le Havre and Cherbourg to their account in New York.^31^

This period came to an end with the invasion of France, the Netherlands, Belgium, and Luxembourg in May 1940. That same week, on May 7, the directors of the National Bank were informed of the delivery of 29 gold bars from the *Reichsbank*. These were also to be shipped across the Atlantic, until menacing rumours of sanctions and blockades on the National Railway in New York reached them. For the time being, it was decided to refrain from overseas shipment and keep the gold in Switzerland.^32^ This meant though, that the gold had to go somewhere else because the bank's evacuation policy did not allow to keep a significant gold stock vaulted in Bern and Zurich. Three days later, Treasurer Blumer filed a declaration for CHF62 for ‘miscellaneous expenses relating to a gold transport from Bern to JMM2’, including lunch for two drivers and five carriers. Three more travel declarations to Interlaken were filed by Blumer on May 23, June 14, and July 24.^33^ The declaration slips do not quantify the gold involved, and simply specify them as ‘large transports’ each involving 2–4 trucks with the help of 5–6 men. With the 3 tons-camion that the bank commonly used for its transports, every delivery could have carried up to CHF50 m. These shipments are typical of the second period of the war, between mid-1940 and mid-1941, when fears of seizing foreign assets by the United States halted the capital flight out of Switzerland and drove the incoming gold instead to the Alps, to the depot in Interlaken.^34^

The bank's feared prophecy came true on 14 June 1941, when the United States froze the assets of neutral European countries. Great Britain had already curtailed the repatriation of Swiss gold reserves held in London, and now the same fate awaited the gold deposit in New York. Soon after the news reached Bern, Treasurer Blumer and his two colleagues left for Interlaken to collect ‘Schweizergold’. A month later, another car returned from the depot loaded with fine gold, and the week after that, two more withanother 6 and 4.5 tons of Swiss bullion. With the restricted access to its foreign accounts, the National Bank was running short of gold and continued reshoring gold by car from Interlaken to Bern in September and October. One of the reasons for this was a lucrative mechanism set up by Swiss commercial banks such as Credit Suisse involving the Portuguese escudo currency.^35^ When the *Reichsbank* ordered goods from Portugal, it paid for these in escudos which it had acquired from Swiss banks in exchange for the looted gold. The Swiss banks had bought these escudos from the Portuguese National Bank in exchange for Swiss francs. The Portuguese then handed these francs in at the Swiss National Bank for Swiss gold. At the end of the day, this resulted in Swiss gold flowing out of the National Bank (hence Blumer's trips to Interlaken) while the reserves of looted Nazi gold in Swiss commercial banks was mounting.^36^

By October 1941, the National Bank had already lost CHF60 m of its gold because of these transactions. It reached out to their congenial contact at the *Reichsbank* to, from that moment onwards, deliver the gold directly to the National Bank instead of trading with the Swiss commercial banks.^37^ This controversial agreement heralded the third period in the dealings with Nazi Germany. Between the fall of 1941 and the end of 1942, the triangular transactions between the Swiss, Portugese, and Germans proliferated, with up to CHF551 m in Nazi gold pouring into the National Bank.^38^ In keeping up with the evacuation policy to avoid large gold holdings in Bern, the bank's directors decided in February 1942 to transfer another CHF100 m of the newly arrived gold to Interlaken, bars and coins that it purchased both from their Axis and Allies trading partners.^39^ Two weeks later, 493 sealed boxes totalling 1957 bars were loaded onto two luggage wagons in Bern for the journey to Interlaken.^40^ At the same time, each of the following months, a car drove in the opposite direction, from Interlaken to Bern to collect small consignments of gold, presumably for shipments to Portugal.^41^ The Portuguese government refused direct gold payments from Germany, but after passing through Swiss hands, the same looted gold, some still stamped with a swastika, or with a seal of the Dutch treasury, was avidly accepted.^42^

But patchily, in the first days of 1943, dark clouds burgeoned around Bern's Bundesplatz where the seats of the National Bank and the federal government stood side by side.^43^ In London, the Allies had issued ‘a formal warning to all concerned, and in particular to persons in neutral countries, that they intend to do their utmost to defeat the methods of dispossession’ practised by Nazi Germany and its trading partners.^44^ Putting pressure on the Swiss government and its banks to show their true colours, the Allies threatened not to recognize the Swiss ownership of looted gold. The warning crossed the news of the Nazi's impending first defeat in Stalingrad. Nevertheless, the Swiss National Bank kept up its defence that the gold purchases from both the Allies and the Axis powers were necessary to maintain the stability of the Swiss franc and that refusing the gold would violate Switzerland's neutrality.^45^ Neutrality did not only refer to military neutrality, but as the Federal Council ascertained, also to the legally unexplored concept of economic neutrality.^46^ Despite this public assertation, that fall, the bank began to sell *Reichbank*-minted coins on the market, an indication of endeavours to dispose of the contested gold of Nazi provenance.^47^ Again, Blumer declared three transports to Interlaken, each time with two large 5-ton trucks, capable of reshoring up to 25 tons of gold to Bern, a figure that roughly corresponds with the CHF106 m it sold on the private gold market that year.^48^ Just two months before the Allies landed in Normandy, the National Bank reported that it ‘had to ward off a veritable flood of gold from the Axis countries’, followed by a drastic reduction of the gold imports from the *Reichsbank*.^49^ Treasurer Blumer continued to make regular visits to Interlaken during these months, five times with three colleagues and four to six local soldiers, for both the delivery and removal of gold.^50^ These visits can be related to the bank's resolution in November 1944 to mint new *Vrenelis*, gold coins with a face value of CHF20, embellished with the young female face of Helvetia posing in front of Alpine summits. At the explicit request of the board, only gold already in the possession of the bank at the end of 1935 was used for the coins.^51^ Why only pre-war gold was to be minted was not explicated, but it suggests an awareness of the disaster that would strike if the coins would have to be recalled, and Helvetia's face turned out to be not so innocent as she appeared.

The moment it came over the wire in Bern that the war in Europe was finally over, the National Bank's mind was already onto something else. The lifting of all evacuation measures left the bank's Governing Board worried: ‘due to the limited space available in the vaults, it is impossible at present to dispense with the use of the evacuation rooms’.^52^ The bank realized that it could impossibly bring all the gold back from the Alps to Bern and Zurich. This spatial parameter presents indisputable evidence that the gold depots in the Alps had not only served as evacuation sites, but that their architectures had instrumentalized the bank's covetous stockpiling of gold. Shortly after this reality check, while the Allies were meeting in Potsdam to plot the postwar peace, the bank hurried to commission the esteemed architect Otto Brechbühl to undertake an extension of the tresors beneath its headquarters in Bern.^53^ A project of this complexity in the old historic centre would take years to realize, so for the time being, the depot in Interlaken remained up and running. Until May 1946, when three nightly explosions killed ten civilians and destroyed large parts of the Fort de Dailly on the other side of the Alps. No one had truly considered the hazards of large, centralized ammunition depots, and in hindsight, situating the gold depot next to five active ammunition depots was rather detrimental.^54^

**Figure 5.**
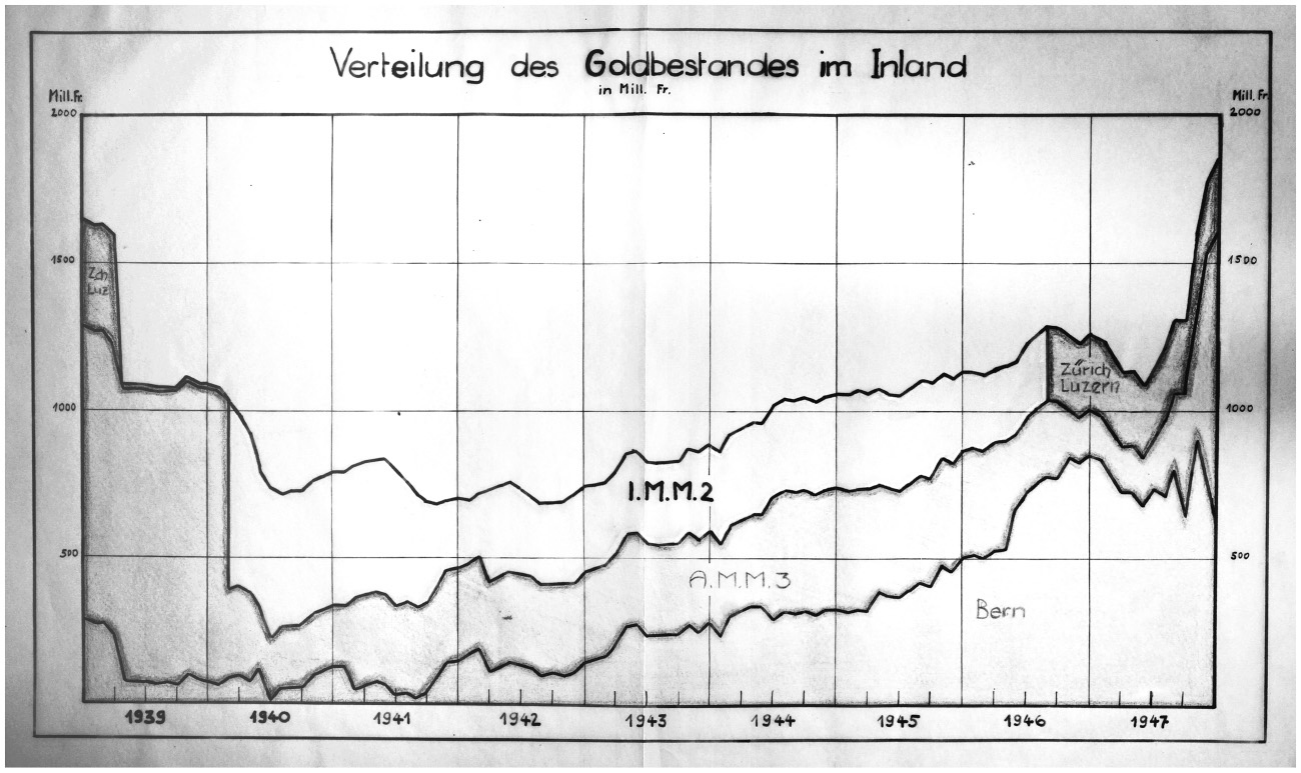
Hand-drawn chart with the domestic distribution of gold stock National Bank between 1939 and 1947. Source: SNB Archive, signature 2.7/2078.

The vacating of the Interlaken depot in 1946 also stands out in a graph (Figure 5) that charts the domestic distribution of the Swiss gold reserves during the war. Hand-drawn by Treasurer Blumer (or one of his assistants), for internal eyes only, it recapitulates a decade in which the spatial and logistical question of storage and transport was deeply intertwined with geopolitical and monetary policies. It has been an centuries-old adage for bankers that money has to roll for the economy to run smoothly, one that was taken quite literally those years, as protecting the national gold meant putting the coins and bars on trains and trucks from one depot to another. The chart shows a changing trend in mid-1940, from a downward line to an increasing value of the bank's gold reserves, requiring additional storage space. This turning point coincided with the armistice of France. The Monday after the collaborationist Petain was installed as France' new Prime Minister, Director Rossy sent out a communiqué to all the bank's departments and branches, informing them that ‘following the French army's laying down of arms, a new situation has arisen on the basis of which the directorate decided today to suspend further evacuation preparations for the time being’.^55^ Almost all evacuation measures taken by the bank (including the transformation of a grand hotel on Lake Thun into an evacuation site for the bank's headquarters) were abolished and the bank reopened its branches so that the economy would be disrupted as little as possible.^56^

At the same time, General Guisan ordered the construction of a fortified Réduit National in the mountainous interior of the country, where the military could retreat, while the rest of the working population was demobilized and put back to work in the factories and fields.^57^ National Bank's Director Schwab backed the plan saying that ‘it is indispensable that those industrial enterprises which are to supply Germany can now dispose of the necessary manpower’.^58^ A diplomatic delegation, including Schwab, was sent to Berlin to successfully negotiate the lifting of Nazi Germany's blockade of imported Swiss goods.^59^ A new defence strategy had been conceived, focussing on economic collaboration instead of military protection of the borders. For the Alpine gold depots, this meant that their purpose effectively changed from an evacuation site protecting against Nazi Germany to a trafficking site catering them. The depots became important cogs in the wheel of the gold trade that bankrolled the war economy of the Axis powers. This changing posture also explains why, when it came to the attention of the Swiss military counterintelligence unit that the site of the Gotthard gold depot was compromised and well-known to Nazi Germany, the General Staff did not order a relocation of the gold.^60^ Deep inside, the down-to-earth bankers, colonels, and politicians must have known that it was not just the Alpine peaks that kept them safe but what lay beneath its tapestry of pastures: the gold of the *Reichsbank*.

Although the contested gold trade between the Swiss banks and Nazi Germany has been extensively documented and discussed by historians since the late 1990s, the material evidence of this infrastructure presented in this article sheds a different light on the involved actors, institutions, and territories. Not only to point out those others complicit in the trade, but more so to complement the quantitative economic histories based on numbers, tables, and graphs with qualitative accounts of people and places, to better understand their interests and actions.^61^ One of these characters is Treasurer Blumer, who possessed a cameleonic ability to change roles from a treasurer managing the assets of the nation to a logistical expert, optimizing timetables and flows of goods between transport and storage, to an engineer drafting plans, ordering ventilation systems and impenetrable armoured doors, and finally to an official with ultimate responsibility for guarding a precious cargo train. These double hats were also the result of close, decades-long partnerships between military, railway companies, and banks. What started as emergency alliances for evacuation in times of war grew into deep entanglements of the public and private sectors. The National Bank's gold was considered the survival insurance of the Swiss monetary system, and its transport, storage, and trade imperative for public interests, even though the shares of the National Bank were ever since its founding for about 45 per cent privately owned by more than 10,000 shareholders.^62^ Shareholders who, at night, out of sight, profited from the bank's parasitizing on the publicly funded infrastructures and bureaucracies of the military and railways. The report on the search for a third evacuation site shows that it was not financially feasible for the bank to pay for a military guard or to build its own depot. It was only by piggybacking on the Reduit infrastructures of the military and the Federal Railways that the bank was able to store the incoming gold, and maintain the trade in Nazi gold. Apart from the modest renovation costs for the gold depot in Interlaken and Blumer's lunch and travel declarations, the financial contribution of the bank was negligible. The history of the Swiss transport, storage, and trading of Nazi gold thus reveals a project built on both pragmatic and symbolic grounds, that was not only part of a national defence strategy, but also for the economic benefit of those who profited from the war and its victims.

## Notes

1 With the exception of the National Bank, the archives of banks in Switzerland are only accessible to the bank's own employees, so that their histories are almost exclusively written by in-house historians. The private archives of Credit Suisse, and for example UBS that recently acquired the bank, were only temporarily opened to independent researchers around 2000, when the Federal Council appointed a commission of historians to study the relations between Switzerland and Nazi Germany during the Second World War. See: J.-F. Bergier, *Switzerland, National Socialism, and the Second World War: Final Report* (Zurich 2002).

2 The note, dated 15 September 1936, was initially addressed to the Federal Department of Finance and later forwarded to the National Bank, suggesting that before this moment, relations between the National Bank and the General Staff were not self-evident. Swiss National Bank Archive, signature 2.7/2078.

3 The Gotthard depot is extensively discussed in the meetings of the National Bank's Governing Board and Banking Committee, see for example: Swiss National Bank Archive. Minutes Meeting of the Bank Committee of the Swiss National Bank, 18/19 May 1937.

4 L. Groen, ‘Das Hotel, das die Credit Suisse rettete’, *Kunst* + *Architektur in der Schweiz* 74, 3 (2023), 4–11.

5 Federal Decree for the purchase of land, dated 14 August 1935. Swiss Federal Archives, signature E27#1000/721#16938*.

6 Letter from the War Material Administration to the Federal Military Department, dated 25 October 1935, concerning the acquiring of the land in Lütscheren. Swiss Federal Archives, signature E27#1000/721#16938*.

7 Newspaper clipping translated from German in *Der Bund*, 328, 17 July 1936. Swiss Federal Archives, signature E27#1000/721#16938*.

8 Report about the incident from the Federal Military Department to the General Staff on 13 July 1936. Swiss Federal Archives, signature E27#1000/721#16938*.

9 Documents by engineers Rothpletz, Lienhard & Cie. from 16 October 1939. Swiss Federal Archives, signature E27#1000/721#16938*.

10 Letter to the General Staff elaborating on the renovation of the Interlaken depots, dated 4 February 1943. Swiss National Bank Archive, signature 1000971.

11 Ibid.

12 Report from Director Rossy to his colleagues about the reconnaissance trip on 15 November 1939. Swiss National Bank Archive, signature 1000971.

13 In an email exchange, the monastery's current prior indeed confirms the retrofit by the cantonal bank in 1939, which was never put into use. Given their humidity, they were hardly usable to store banknotes or security papers, only the non-corroding gold would survive, the prior assured.

14 Report from Director Rossy to his colleagues about the reconnaissance trip on 15 November 1939. Swiss National Bank Archive, signature 1000971.

15 Document dated November 16, 1939. Swiss National Bank Archive, signature 1000971.

16 Report from Director Rossy to his colleagues about the reconnaissance trip on 15 November 1939. Swiss National Bank Archive, signature 1000971.

17 Ibid.

18 Swiss Federal Archives, signature J1.238#1994/175#1*

19 Letter from Colonel Rathgeb to Colonel and Director Schwab of the National Bank, dated 19 November 1939. Swiss National Bank Archive, signature 1000971. For Bern's snow report of that winter see: Meteoswiss, ‘Annual date of the first measurable snow cover in the winter periods from 1931/32 onwards,’ 2023.

20 Based on telegram exchange between Director Schwab and Treasurer Blumer on 15 January 1940. Swiss National Bank Archive, signature 1000971.

21 Correspondence between the bank and the Army Staff on 24 and 25 January 1940. Swiss National Bank Archive, signature 1000971.

22 Transfer report by Treasurer Blumer to the Governing Board, dated 6 February 1940. Swiss National Bank Archive, signature 1000971.

23 Monika Dommann, ‘Be Wise – Palletize: die Transformationen eines Transportbretts zwischen den USA und Europa im Zeitalter der Logistik’, *Traverse: Zeitschrift für Geschichte* 16 (2009), 24.

24 Transfer report by Treasurer Blumer to the Governing Board, dated 6 February 1940. Swiss National Bank Archive, signature 1000971.

25 Ibid.

26 Ibid.

27 S. E. Eizenstat and W. Z. Slany, *U.S. and Allied Efforts To Recover and Restore Gold and Other Assets Stolen or Hidden by Germany During World War II. Preliminary Study *(Washington 1997), iv.

28 Bergier, *Switzerland, National Socialism, and the Second World War: Final Report*, 185, 248.

29 The mentioned paper divides the Second World War in the following five periods: a first period from the outbreak of War until 2Q 1940 (1); the second period from 3Q 1940 until 3Q 1941 (2); the third period from 4Q 1941 until end-1942; the fourth period from 1Q 1943 until 1Q 1944; and the fifth period from 2Q 1944 until the end of the War. See V. Crettol and P. Halbeisen, *Monetary policy background to the gold transactions of the Swiss National Bank in the Second World War* (Zurich 1999), 18–21.

30 J. Tanner, *Geschichte der Schweiz im 20. Jahrhundert* (Berlin 2015), 254.

31 Crettol & Halbeisen, *Monetary policy background to the gold transactions of the Swiss National Bank in the Second World War*, 18–21.

32 Swiss National Bank Archive. Minutes Meeting of the Governing Board of the Swiss National Bank, 7 May 1940.

33 Swiss National Bank Archive, signature 1000971.

34 Crettol & Halbeisen, *Monetary policy background to the gold transactions of the Swiss National Bank in the Second World War*, 22.

35 J. Jung, *Zwischen Bundeshaus und Paradeplatz: Die Banken der Credit Suisse Group im Zweiten Weltkrieg* (Zurich 2001), 225–6.

36 Crettol & Halbeisen, *Monetary policy background to the gold transactions of the Swiss National Bank in the Second World War*, 25.

37 Swiss National Bank Archive. Minutes Meeting of the Governing Board of the Swiss National Bank, 2 October 1941.

38 Crettol & Halbeisen, *Monetary policy background to the gold transactions of the Swiss National Bank in the Second World War*, 28, 31.

39 Swiss National Bank Archive. Minutes Meeting of the Governing Board of the Swiss National Bank, 19 February 1942.

40 Letter with instructions to the command of JMM2 regarding the upcoming transport, dated 28 February 1942, and report of the completed transport dated 9 March 1942. Swiss National Bank Archive, signature 1000971.

41 These transports took place on 23 March, 27 April, 4 June, 17 July. Swiss National Bank Archive, signature 1000971.

42 Fifty years later a few bars embossed with swastika were still vaulted in Lisbon. See Marlise Simons, ‘Nazi Gold and Portugal's Murky Role,’ *The New York Times* (10 January 1997).

43 For an elaboration on the changing role of the Swiss banks in the trade of Nazi gold, see also: Arthur L. Smith Jr., *Hitler's Gold. The Story of the Nazi War Loot* (Oxford 1996), 48–67.

44 Quote from Inter-Allied Declaration Against Acts of Dispossession Committed in Territories Under Enemy Occupation or Control issued on 5 January 1943. See: United States Department of State, Foreign Relations of the United States: Diplomatic Papers Volume 1 (Washington 1943), 444.

45 Bergier, *Switzerland, National Socialism, and the Second World War: Final Report*, 252.

46 Speech from Swiss president Walther Stampfli in May 1944.

47 Crettol & Halbeisen, *Monetary policy background to the gold transactions of the Swiss National Bank in the Second World War*, 33, 35.

48 Announcements of visits to the command of JMM2 on 30 September and 12 November 1943 and 3 January 1944. Swiss National Bank Archive, signature 1000971. See also: Crettol & Halbeisen, *Monetary policy background to the gold transactions of the Swiss National Bank in the Second World War*, 35.

49 Crettol & Halbeisen, *Monetary policy background to the gold transactions of the Swiss National Bank in the Second World War*, 36.

50 These visits took place on 9 October and 21 December 1944 and 5 January, 15 February, and 1 March 1945. Swiss National Bank Archive, signature 1000971.

51 Swiss National Bank Archive. Minutes Meeting of the Governing Board of the Swiss National Bank, 9 November 1944.

52 Swiss National Bank Archive. Minutes Meeting of the Governing Board of the Swiss National Bank, 28 June 1945.

53 Swiss National Bank Archive. Minutes Meeting of the Governing Board of the Swiss National Bank, 24 July 1945.

54 Swiss National Bank Archive. Minutes Meeting of the Governing Board of the Swiss National Bank, 17 July 1946.

55 Swiss National Bank Archive. Letter by Director Rossy to all departments, branches, and agencies, 17 June 1940.

56 In the spring of 1940, the National Bank started transforming a grand hotel and castle in Spiez into an evacuation site for its headquarters, see: *Groen, Kunst + Architektur in der Schweiz*, 4–11. For the abolishing of evacuation measures see: Swiss National Bank Archive. Minutes of the Governing Board of the National Bank, 16 September 1940.

57 Tanner*, Geschichte der Schweiz im 20. Jahrhundert*, 267–8.

58 J. Tanner, ‘Réduit National und Aussenwirtschaft: Wechselwirkungen zwischen militärischer Dissuasion und ökonomischer Kooperation mit den Achsenmächten,’ in Philip Sarasin, Regina Wecker (eds) *Raubgold, Reduit, Flüchtlinge: Zur Geschichte der Schweiz im Zweiten Weltkrieg* (Zurich 1998), 97.

59 Confidential letter from Director Schwab to the president of the Swiss confederation Pilet-Golaz, dated 13 August 1940, quoted in: Tanner, *Raubgold, Reduit, Flüchtlinge,* 97.

60 In February 1942, the Swiss military intelligence investigated the case of traitor Jakob Meier who revealed the location of the Gotthard depot to Nazi Germany, by then the General Staff was aware that the gold depot was compromised. Swiss Federal Archives, signature E4800.7#1995/20#5*.

61 This methodology builds upon Carlo Ginzburg's plea for the writing of micro-histories and privileging a single detailed account over a multiplicity of generalised abstractions, see: C. Ginzburg, ‘Clues: Roots of an Evidential Paradigm,’ in *Clues, Myths, and the Historical Method* (Baltimore 2013), 96–125.

62 Since its founding the shares of the Swiss National Bank were privately owned for about 45 per cent with more than 10,000 private shareholders. See: Swiss National Bank, *The Swiss National Bank 1907–2007* (Zurich 2007), 517–21.

